# Responding to the health needs of survivors of human trafficking: a systematic review

**DOI:** 10.1186/s12913-016-1538-8

**Published:** 2016-07-29

**Authors:** Stacey Hemmings, Sharon Jakobowitz, Melanie Abas, Debra Bick, Louise M. Howard, Nicky Stanley, Cathy Zimmerman, Sian Oram

**Affiliations:** 1 King’s College London, David Goldberg Centre, De Crespigny Park, London, SE5 8AF UK; 2 King’s College London, James Clerk Maxwell Building, Waterloo, London, SE1 8WA UK; 3 University of Central Lancashire, School of Social Work, Harrington Building, Preston, PR1 2HE UK; 4 London School of Hygiene & Tropical Medicine, Keppel Street, London, WC1E 7HT UK

**Keywords:** Systematic review, Qualitative, Human trafficking, Violence

## Abstract

**Background:**

Despite the multiple physical and psychological health consequences associated with human trafficking, there is little evidence-based guidance available for health providers on assessing and meeting the health needs of trafficked people. We aimed to review literature that provided guidance or research on care provision for people who had been trafficked.

**Methods:**

We conducted a systematic review and qualitative analysis of peer-reviewed and grey literature. Data sources included electronic databases, reference list screening, citation tracking, and expert recommendations. Documents were included if they reported on: 1) male or females (adults or children) who were currently or had previously been trafficked; 2) health interventions or service provision; 3) primary, secondary, tertiary or specialist post-trafficking services; and 4) World Bank high income countries. Two reviewers independently screened and quality appraised documents. Framework analysis was used to analyse extracted data.

**Results:**

Forty-four documents were included, 19 of which reported findings of primary studies and nine of which exclusively addressed children. Evidence to inform the identification, referral and care of trafficked people is extremely limited. Within current literature on survivor identification, key indicators included signs of physical and sexual abuse, absence of documentation, and being accompanied by a controlling companion. Findings highlighted the importance of interviewing possible victims in private, using professional interpreters, and building trust. For provision of care, key themes included the importance of comprehensive needs assessments, adhering to principles of trauma-informed care, and cultural sensitivity. Further prominent themes were the necessity of multi-agency working strategies and well-defined referral pathways.

**Conclusions:**

Human trafficking survivors require healthcare that is trauma-informed and culturally sensitive to their particular needs. Coordination is needed between health providers and statutory and voluntary organisations. Future research should generate empirical evidence to develop trafficking indicators for use by health providers, alongside validated screening tools, and evaluate the effectiveness of psychological interventions.

**Electronic supplementary material:**

The online version of this article (doi:10.1186/s12913-016-1538-8) contains supplementary material, which is available to authorized users.

## Background

Human trafficking, which is estimated to affect 20.9 million people worldwide, is a serious crime and a violation of human rights [1]. It involves the recruitment and transportation of people – often by the use of force, fraud, deception or coercion – for the purposes of exploitation [2]. People may be exploited for forced sex work, domestic servitude, forced labour in industries such as construction, agriculture, fishing, factory labour, and in forced criminal activity.

Although evidence on health and human trafficking is limited, a systematic review found that human trafficking is associated with high levels of physical and sexual violence prior to and during trafficking and a range of health problems in the post-trafficking period [3]. Studies with survivors identified high prevalence of depression, anxiety and post-traumatic stress disorder and symptoms such as headache, fatigue, dizziness, and back and stomach pain [3–5]. A number of policy and guidance documents describe assistance measures to respond to the needs of trafficked persons, which include medical and psychological care [6, 7], yet there still appears to be little evidence-based guidance available on how to plan, assess or provide for the health needs of trafficked adults and children. Healthcare professionals believe they have insufficient knowledge and lack confidence about how to respond appropriately to the needs of trafficked people [8, 9]. Recent research in health organisations in areas with high numbers of trafficking victims identified by police in England, suggest that 13 % of health professionals had been in contact with a person that they suspect has been trafficked [8]. Such findings underlie the importance of professionals increasing their preparedness to identify potential cases of human trafficking and make appropriate referrals.

This review synthesises evidence on current knowledge and practice in responding to the health needs of trafficked people, specifically exploring identification, referral and provision of care by the healthcare sector.

## Methods

### Study design

Systematic review. The review follows Preferred Reporting Items for Systematic Reviews and Meta-Analyses (PRISMA) reporting guidelines (for the PRISMA checklist, see Additional file 1).

### Data sources

Sixteen biomedical and social science databases (including MEDLINE, Embase and PsycINFO) and 21 grey literature websites and databases (including Department of Health in England; Open Grey; La Strata International and Innocenti project) were searched from 1^st^ January 1990 to February 2015 (see Additional file 2 for a full list of databases used). A combination of controlled vocabulary index and free text terms were used to search electronic databases, including terms relating to human trafficking (e.g. “human trafficking”, “people trafficking”, “sex trafficking”, “trafficked people”), health services (e.g. “health services/”, “hospital”, “clinic”, “family planning”, “emergency”, “psychol*”, “psychiatry*”), health personnel (e.g. “health personnel/”, “clinician”, “doctor”, “health professional”, “nurs*”), and care approaches and interventions (e.g. “identif*”, “respons*”, “intervene*”, “prevent*”, “treat*”); see Additional file 3 for a full list of search terms used. Where a controlled vocabulary index did not exist for a database or website, only free text terms were used. Electronic searches were supplemented using reference list screening, forwards citation tracking using Web of Science and Google Scholar, and expert recommendations. All identified references were imported to EndNote.

### Selection criteria

Documents were eligible for inclusion in the review if they: 1) addressed adults and/or children who were currently or had previously been trafficked; 2) reported on health interventions or service provision;3) focused on primary, secondary, tertiary or emergency health settings, specialist post-trafficking support services in either the statutory and voluntary sectors, or statutory, voluntary and private social care settings; and 4) reported on World Bank high income countries. Peer-reviewed and grey literature (e.g. theses, dissertations, published or unpublished reports) were included and no language restrictions were used. Editorials, opinion pieces, and textbooks were excluded from the review. Human trafficking was defined in accordance with the United Nations Optional Protocol on the Prevention, Punishment and Suppression of Trafficking in Persons, Especially Women and Children [2].

### Screening

Two reviewers (LN and SH) independently screened titles and abstracts for eligibility. Following abstract screening, two reviewers (LN and SH) assessed the full texts of potentially eligible studies. Uncertainty or disagreement was resolved by consensus decision. If a decision could not be made, a third reviewer was consulted (SO). If studies collected relevant data but did not report it, data were requested from the authors. Details of the excluded papers and reasons for exclusion are available on request.

Data extraction and quality appraisal: Data were extracted from each study into a standardised extraction form. Data extraction was divided between two reviewers (SJ and SH). As a check, each reviewer independently extracted data from a random 25 % of papers assigned to the other; no differences were found. Data were extracted on: a) study populations and sample characteristics: b) study design and methods: and c) data relating to: (i) trafficked people’s understandings; expectations and experiences of their health needs/health sector; (ii) care approaches or interventions used to assist health providers in identifying cases of human trafficking; (iii) care approaches or interventions used to assist health providers in responding to cases of human trafficking; and (iv) regional, national and international policies implemented to promote the identification, referral and provision of care to trafficked people. Twenty-five percent of all screenings and full texts were checked for agreement and consistency.

The methodological quality of studies was independently assessed by two reviewers (SH and SJ) using checklists adapted from the Joanna Briggs Institute and varied by type of document (see Additional file 4 for full quality appraisal checklists) [10]. Complete listings of all studies and quality appraisal scores are presented in Additional file 5: Table S1.

### Analysis

Extracted data were analysed by two reviewers (SH and SJ) using framework analysis [11] - a matrix-based method involving the construction of thematic groupings into which data can be categorised [12]. The benefits of framework analysis have been documented in previous systematic reviews [13, 14] and have included its rigour, as well as its flexibility in allowing multiple members of the research team to be involved in analysis [15]. As such, analysis began with immersion and familiarisation through repeated reading of the extracted data. The original texts were frequently referred to in order to check consistency and accuracy of the text as well as the emerging concepts and themes [16]. Concepts were charted onto a Microsoft Excel spreadsheet in order to visualise overarching themes and to draft a working conceptual framework [16]. The conceptual framework was iteratively revised as new categories emerged and previous categories were merged.

## Results

**Fig. 1:**
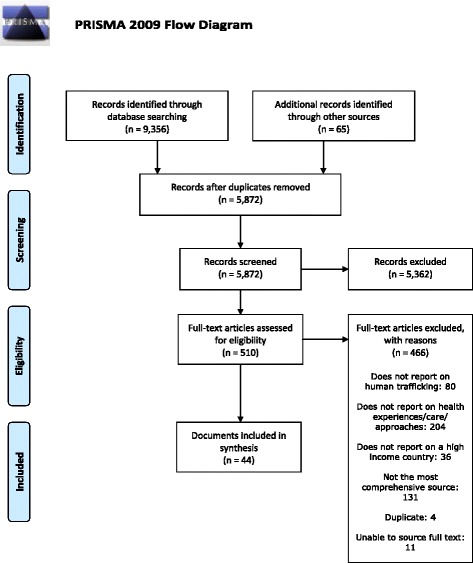
PRISMA 2009 Flow Diagram

As shown in Fig. 1, 44 papers were included in the review. Searches identified 9,356 documents for title and abstract screening and 510 documents for full text screening. Full text papers included 18 peer-reviewed journal articles, three conference poster presentations, 20 published reports, two unpublished dissertations, and one unpublished report. Of the 44 included papers, 19 reported the findings of primary studies. Three authors were contacted with a request for additional data; only one author was able to provide additional information [17].

The key themes that emerged from the literature concerned: promoting disclosure; providing care; ensuring safety; supporting recovery; working in partnership; and developing services. Each is discussed in detail below.

### Promoting disclosure

#### Identification

Seventeen papers described potential indicators of exploitation and suggested that healthcare professionals could play a role in the identification of victims of human trafficking [17–33]. Commonly reported indicators covered a wide range of symptoms and behaviours and included signs of physical and sexual abuse (*n* = 16 papers and *n* = 14 papers, respectively), such as broken bones, burns, chronic pelvic pain and sexually transmitted infections [17–29, 31–33], as well as the inability to speak the local language (*n* = 8) [19, 20, 22, 24–27, 29, 33], absence of official documents (*n* = 10) [18, 23–26, 28, 31, 33], fear of deportation (*n* = 5) [21, 28, 31–33], inconsistencies in presentation (e.g. dates, names and addresses) (*n* = 9) [17, 21, 22, 24, 25, 28, 29, 31, 33, 34], and attending with/accompanied by a seemingly controlling companion (*n* = 12) [17, 18, 20–22, 24, 26, 27, 29, 31, 33]. Additional indicators for children included unusually high levels of maturity and self-confidence (*n* = 3) [21, 28, 31], access to money and goods that are inconsistent with the child’s age (*n* = 4) [17, 21, 28, 31], not living with parents or relatives (*n* = 4) [21–23, 31] and not attending school (*n* = 6) [17, 21, 22, 28, 30, 31]. Additional file 6: Table S2 summarises the reported indicators.

#### Privacy

Studies reporting on the healthcare experiences of trafficked people found that many victims had come into contact with health services but had been seen in the presence of their traffickers, preventing disclosure [17, 19, 20, 24, 27]. Several papers highlighted the importance of seeing suspected victims privately, even if the person accompanying them claimed to be a friend or family member [17–20, 24, 27, 29, 32–35], with one suggesting the applicability of domestic violence protocols in this regard [18].

#### Interpreters

The importance of providing access to interpreters, and of never allowing an individual accompanying a possible victim to interpret for them, was noted by several papers [18, 20, 24, 25, 27, 29, 32, 33, 35–38]. Authors argued that access to a professional interpreter could facilitate the disclosure of victims’ experiences, help build trust, and support the trafficked person in navigating health services [24, 27, 36, 38]. Papers also highlighted, however, the potential difficulties of working with interpreters, including ensuring confidentiality, problems of stigma and embarrassment and accessing interpreters experienced in providing trauma informed services and fluent in patients’ preferred languages and dialects [22, 24, 29, 36–38]. Zimmerman et al. [36], noting the importance of interpreting not just patients’ language, but also their social and cultural circumstances, suggested the use of cultural mediators (i.e. persons sharing the same customs and language of the victim), where available. Expanding on this in later work, Zimmerman warned that persons from the same village or local community (either in the home or destination location) should not be permitted to interpret for a trafficked individual, as this may inhibit the individual from speaking freely and information provided may turn into gossip or otherwise stigmatise them [32]. The Family Violence Prevention Fund [24] also cautioned that some victims of trafficking may feel embarrassed to talk about stigmatising subjects with professionals from the same cultural background.

#### Building trust

The importance of building trust with trafficked people was a recurrent theme across papers [22, 24, 25, 27, 29, 32, 36–42], with several describing how trafficked people often experience intense feelings of betrayal, guilt, and shame [27, 36, 42]. Authors suggested that healthcare professionals should use sensitive, informal and non-judgmental language and acknowledge victims’ possible fears about the consequences of disclosing their experiences [22, 27, 32, 36, 42]. Authors also recommended using open-ended questions and, in order to reduce the risk of re-traumatisation, ensuring that only relevant questions are asked [22, 32, 39]. Authors similarly indicated that trafficked people may need both longer appointment times and a longer overall duration of contact with healthcare services in order to build trusting therapeutic relationships [22, 38].

### Providing care

#### Comprehensive needs assessment

Several papers described the importance of providing comprehensive needs assessments to identify the multiple health and social care needs [6, 25, 26, 36, 37, 41, 43–46]. The provision of a prompt response to trafficked people’s immediate needs, including their physical health, mental health, safety and accommodation was emphasised by several papers [25, 33, 37–39]. Two papers also highlighted the importance of needs assessments to identify potential longer-term health problems, as well as immediate needs [39, 42], and for assessments to be repeated as needs changed over time [25, 42].

#### Ensuring informed consent

The importance of informed consent was emphasised in a number of papers, including guidance published by the International Organisation for Migration, European Union, and the Department of Health in England [6, 8, 22, 32, 37, 42], particularly with regards to medical examinations and Human Immunodeficiency Virus (HIV) testing. Although informed consent is a fundamental component of all medical practice, papers highlighted the coercive and involuntary nature of the trafficking experience for victims and as such the use of informed consent as an avenue to facilitate dignity and control [32, 47]. Informed consent was identified as a fundamental component of trauma-informed care (see next section) and authors argued that to be meaningful, consent procedures must include the provision of information at a time and in ways that are appropriate for each survivor for example in a language that they understand or by accommodating problems with literacy [32]. In this way, the process can contribute to improved survivor autonomy and engagement [42].

#### Trauma-informed care

Several papers emphasised the importance of following a trauma-informed approach when caring for trafficked people [18, 37, 38, 41, 42, 46, 48]. Trauma-informed care includes a commitment to empowerment [37, 42, 46] and victim safety [37, 38, 42, 46] and recognises the impact of multiple traumatic events across the individual’s life-course [24]. Some authors highlighted the importance of avoiding re-traumatisation, for example by ensuring that survivors are not pressured to discuss the detail of their events before they are ready [1, 38].

#### Culturally sensitive working

The importance of developing culturally appropriate and competent services for trafficked people was another common theme across the reviewed papers [18, 19, 24, 25, 27, 37–39, 41–43, 49]. Borland & Zimmerman [32] (page 41) described culturally sensitive care as “the provision of care that is attentive to the various ways people from diverse backgrounds experience and express illness and how they respond to care”. In particular, authors drew attention to cultural differences in attitudes towards health, particularly mental health, and highlighted that typically Western approaches, such as counselling, may not be appropriate for this client group [43]. In qualitative work by Aron et al. [39], victims described wanting other services, outside of one-to-one therapy, to address their emotional needs such as acupuncture. Victims described their experiences of one-to-one therapy as often shameful and blaming, and they found that western-style talk therapy did not always resonate with their cultural backgrounds [37, 39].

### Ensuring safety

#### Threat of violence

The potential risk of harm to trafficked patients and to staff involved in their care was mentioned in four documents [8, 18, 32, 34, 35, 37, 49, 50]. For example, Borland & Zimmerman [32] explained that “in some cases, individuals who have escaped traffickers may be pursued by them or their co-conspirators, especially if the trafficked person is participating in a criminal investigation against the trafficker”. Additionally, a report by the Department of Health in England suggested healthcare professionals feared being placed at risk of violence through their work with trafficked people [8], and both this report and guidance from the International Organisation for Migration recommended that health services should ensure security guidance and procedures are in place to safeguard both trafficked individuals and staff [8, 32].

#### Confidentiality

The importance of patient confidentiality was emphasised by several papers, including maintaining the security and confidentiality of patient records and ensuring robust information-sharing protocols [8, 18, 32, 34, 35, 37, 49]. One author noted that the environment in which patients are seen should be a confidential space in which the individual feels safe to share traumatic memories [49]. Attention was also drawn to the limits of confidentiality, highlighting the need for awareness of and compliance with local reporting requirements, including mandatory reporting of child maltreatment [18]. Borland & Zimmerman [32] also drew attention to confidentiality in respect of police referrals and argued that calling police or immigrations services should be avoided unless the victim has provided consent. The authors argue that victims may have “well-founded reasons to avoid authorities” (page 29). However if it is necessary to call police services, then this should be discussed clearly with the victim, and a referral made to a specific trusted police contact point where possible [32].

### Supporting recovery

#### Psychological therapies

No primary research was identified that reported on the efficacy of suggested therapeutic interventions for victims of trafficking or the potential to adapt various therapies for survivors of domestic violence and sexual assault or refugees, such as, for example, cognitive behavioural therapy (CBT), trauma-focused CBT (TF-CBT), peer-support and psycho-education [18, 20, 24, 36, 38, 49, 50]. Brief psychotherapy has been recommended by the International Organisation for Migration and one author reported having used psychodrama therapy in a group setting with trafficking victims [1, 43, 45, 51]. The potential value of adjunctive therapies, such as mindfulness, art therapy, writing, music and outdoor activities, was also mentioned for further exploration [38, 43, 48]. However, although authors acknowledged possible similarities between trafficked people and other populations exposed to traumatic events, authors also highlighted important differences. For example, one review suggested that group therapy might be less helpful for survivors of human trafficking than for survivors of domestic violence, because trafficked people may have been asked to participate in the abuse of others [49].

### Working in partnership

#### Multi-agency working

Papers emphasised the importance of working closely with other agencies such as law enforcement and social services [8, 18, 21, 23, 24, 26, 29, 32, 41, 52–54]. This was seen as integral to providing victims with the support and continuity of care needed to engage with both immediate and longer-term recovery goals [21, 34]. Authors called for better and more efficient collaboration between services, mentioning the poor information sharing, communication, and partnership arrangements as barriers to coordinating a victim’s care between several organisations [8, 23, 26]. However whilst calling for greater coordination, authors acknowledged that health professionals may have concerns about possible legal ramifications for the victim if they were to report their suspicions to other statutory agencies [32, 33]. Authors also reported poor knowledge of trafficked people’s rights and entitlements among healthcare professionals, and suggested this was likely to hinder coordination with other services [26, 55].

#### Developing referral pathways

Papers emphasised the need for healthcare services to establish clear referral pathways to ensure appropriate support and continuity of care for trafficked people [8, 36]. Several authors noted that referral pathways for child victims of trafficking were generally more clearly delineated than those for adult victims [7, 21, 28, 31, 53]. Referral pathways were particularly poorly specified with respect to adults with no recourse to public funds [8, 36].

### Developing services

#### Models of care

Several papers reported that specialist services for survivors of trafficking were lacking, and advocated the development of various specific service models, including one-stop-shops, mobile outreach and trauma-specific services [18, 41, 42, 48, 56]. It was suggested that one-stop shops, could assist victims to receive comprehensive needs assessment and timely referrals, while outreach services might ameliorate access difficulties for some victims. Two papers highlighted the importance of providing female-only services due to the need for sensitivity around asking female survivors of human trafficking to speak about sexual health problems, in particular to male doctors or interpreters [36, 56].

## Discussion

To our knowledge, this is the first review to synthesise information on identifying and responding to human trafficking in healthcare settings. The review highlights the lack of empirical evidence to support the identification, referral, and care of victims of trafficking in healthcare settings and identifies priorities for future research in this area. However, analysis of the available literature suggests a general consensus regarding important aspects of care, for example, the benefits of comprehensive, tailored, and continuous support for survivors of trafficking from the point of identification through to long term recovery, and an emphasis on trauma-informed, culturally appropriate care. Table 1 details our recommendations for healthcare providers based on our analysis and synthesis of the literature. Recommendations address the steps providers should take to improve preparedness to respond, disclosure, immediate responses and ongoing care.

**Table 1:** Recommendations for response to human trafficking victims

Points of contact	Recommendations for service providers
Promoting Disclosure	Attend training on human trafficking. Be aware of identifiers. Develop safety protocols for patients and staff. Have awareness of referral pathways.
Initial Contact	Create a private space for patient interaction. Be non-judgemental and sensitive. Be aware of issues around confidentiality. Provide choice regarding the gender and cultural background of interpreter.
Immediate responses to disclosure	Ask case relevant questions. Gain informed consent. Follow protocols created for similar populations. Carry out comprehensive needs assessments. Provide holistic care addressing health and social care needs. Employ trauma-focused approaches. Offer longer appointments.
Ongoing responses	Offer victims longer contact with services. Frequently review victim’s needs, as these will change over time. Engage in multi-agency working and information sharing. Recognise the value of adjunctive therapies. Develop specific service models for victims of trafficking.

### Identification of trafficked people

The review highlights several indicators that might suggest to healthcare professionals that their patient may be a victim of trafficking, including signs of abuse and neglect, unfamiliarity with the local language, being accompanied by a seemingly controlling companion and a lack of official documentation. There is potential for such indicators to inform the development of an easy to use checklist for frontline practitioners. Moreover, the review identified options to help potential victims disclose their situations, such as finding a private space for a consultation, taking time to gain trust and avoiding the use of companions or those accompanying the possible victim as interpreters. Yet, at the same time, it also suggests the range of potential concerns that providers might have about making these types of inquiries, including risks to patient and provider safety and the potential legal consequences for the victim of information sharing. These potential hindrances will need to be addressed by health policymakers in conjunction with the wider post-trafficking support network to enable and encourage providers to engage in an identification and referral process. Existing good practice guidelines for working with victims of domestic and sexual violence may be instructive in this regard [57, 58].

### Care for survivors of human trafficking

Trafficked people may present to healthcare services with multiple physical, psychological, and social care needs. Responding to these needs requires that healthcare professionals adopt trauma-informed and culturally-sensitive approaches to working with victims of trafficking, conduct comprehensive health assessments, and collaborate with a range of agencies, including law enforcement and voluntary support services. Healthcare professionals’ ability to provide care and to refer for further support is likely to be affected by the provision of temporary or permanent legal residency (‘leave to remain’) to survivors of human trafficking and the availability of stable housing, financial, and legal support [59]. Training for healthcare professionals should include information about in-country referral and support options for trafficked people and national reporting requirements, if applicable. At the local level, healthcare professionals should establish clear referral pathways and information-sharing protocols with relevant agencies.

Studies have highlighted the high prevalence and enduring nature of mental health problems among survivors of human trafficking in contact with support services [3, 4]. It is likely that psychological interventions to promote the recovery of trafficked people will need to take account of physical and sexual abuse during - and often prior to – trafficking and to work to stabilise physical and psychological health and to address social needs before commencing trauma-focused therapy [4]. However, no studies were identified that tested the effectiveness of psychological interventions for trafficked people. The acceptability of evidence-based treatments for post-traumatic stress disorder (PTSD) and depression – such as cognitive behavioural therapy, narrative exposure therapy, and eye movement desensitization and reprocessing – among trafficked people is uncertain, as is the generalizability of therapies effective for other traumatised groups such as victims of domestic violence and asylum seekers and refugees. Research to investigate the efficacy of psychological interventions for survivors of human trafficking is urgently needed.

### Strengths and Limitations

The review used a comprehensive search strategy (including electronic searches, reference list screening, citation tracking and expert recommendations) and robust methodology to screen, extract, and analyse data. Data were synthesised using framework analysis, described in previous systematic reviews as useful in healthcare policy syntheses [12, 14]. However, the review is limited by a lack of evidence from primary studies. Furthermore, as studies predominantly referred to female or child victims of trafficking, limited conclusions can be drawn regarding best practice in responding to the healthcare needs of male victims. The review was restricted to materials reporting on high-income countries, and findings may not be generalizable to low- and middle-income country settings.

### Implications for future research

This review identified several priorities for future research. Potential indicators of human trafficking have been described but are not underpinned by empirical evidence; research is needed to refine these indicators and to develop and test the sensitivity and specificity of screening tools to identify victims of human trafficking in healthcare settings. Research is also needed to evaluate the effectiveness of training programmes to improve not only healthcare professionals’ knowledge and understanding of human trafficking, but also the identification of victims and appropriate referral and care. Evidence on victims’ experiences and expectations of healthcare, including mental healthcare, is urgently needed to inform the development of resource and education packages, improve service provision, and investigate the generalizability of guidelines for working with victims of violence and with vulnerable migrants to this population. Crucially, in the face of strong evidence of high prevalence of depression and PTSD among victims of trafficking, experimental studies are urgently needed to test the acceptability and effectiveness of psychological interventions to support the recovery of this vulnerable population.

## Conclusions

Fundamentally, human trafficking is a criminal form of extreme exploitation and abuse, from which individuals suffer multiple physical, psychological, and sexual and reproductive health problems. To foster recovery from this crime, healthcare professionals must be at the centre of responses for survivors. Responding to survivors’ needs requires that healthcare professionals adopt trauma-informed and culturally-sensitive approaches, conduct comprehensive health assessments, and participate in a reliable referral network, including law enforcement and voluntary support services. Training for healthcare professionals should include information about in-country referral and support options for trafficked people and national reporting requirements, if applicable. At the local level, healthcare professionals should establish clear referral pathways, trustworthy points of contact and information-sharing protocols with relevant agencies. Further health services research is urgently needed to enable health professionals to fully engage in identifying, referring and caring for victims of trafficking.

## Abbreviations

CBT, cognitive behavioural therapy; HIV, human immunodeficiency virus; PRISMA, preferred reporting items for systematic reviews and meta-analyses; PTSD, post-traumatic stress disorder; TF-CBT, trauma focused cognitive behavioural therapy

## Additional files

Additional file 1: PRISMA Checklist. (DOCX 19 kb) Additional file 2: Details of databases searched for the review. (DOCX 15 kb) Additional file 3: Search terms used. (DOCX 15 kb) Additional file 4: Quality appraisal checklist. (DOCX 15 kb) Additional file 5: Table S1. Characteristics and quality scores of included studies. (DOCX 28 kb) Additional file 6: Table S2. Potential Identifiers for Adult and Child Victims of Human Trafficking. (DOCX 30 kb)

## References

[1] International Labour Office, ILO Global Estimate of Forced Labour: Results and Methdology, International Labour Office, 2012: Geneva.

[2] United Nations, Protocol to Prevent, Suppress and Punish Trafficking in Persons, Especially Women and Children, Supplmenting The United Nations Convention Against Transnational Organised Crime. New York: United Nations; 2000.

[3] Oram S (2012). Prevalence and risk of violence and the physical, mental, and sexual health problems associated with human trafficking: systematic review. PLoS Med.

[4] Abas M, et al. Risk factors for mental disorders in women survivors of human trafficking: a historical cohort study. BMC Psychiatry 2013:13(3):204. doi: 10.1186/1471-244X-13-204. 10.1186/1471-244X-13-204 PMC3737054 23914952

[5] Kiss L (2015). Health of men, women, and children in post-trafficking services in Cambodia, Thailand, and Vietnam: an observational cross-sectional study. Lancet Glob Health.

[6] European Parliament, Directive 2011/36/EU of the European parliament and of the council of 5 April 2011 on preventing and combating trafficking in human beings and protecting its victims, and replacing Council Framework Decision 2002/629/JHA, 2011. Brussels.

[7] Taskforce on the Health Aspects of Violence Against Women and Children, Report from the Harmful Traditional Practices and Human Trafficking sub-group: Responding to violence against women and children – the role of the NHS. 2010.

[8] Ross C (2015). Human Trafficking and Health: A Cross-Sectional Survey of NHS Professionals’ Contact with Victims. BMJ Open.

[9] Viergever RF, et al. Health care providers and human trafficking: what do they know, what do they need to know? Findings from the Middle East, the Caribbean, and Central America. Frontiers. 2015:3(6). doi: 10.3389/fpubh.2015.00006. eCollection 2015. 10.3389/fpubh.2015.00006 PMC4310216 25688343

[10] Joanna Briggs Institute, Joanna Briggs Institute Reviewers’ Manual 2011 Edition. Australia: University of Adelaide; 2011.

[11] Noblit GW, Hare RD (1988). Meta-Ethnography: Synthesing Qualitative Studies.

[12] Dixon-Woods M (2011). Using framework-based synthesis for conducting reviews of qualitative studies. BMC Med.

[13] Oliver SR (2008). A multidimensional conceptual framework for analysing public involvement in health services research. Health Expect.

[14] Carroll C, Booth A, Cooper K (2011). A worked example of “best fit” framework synthesis: A systematic review of views concerning the taking of some potential chemopreventive agents. BMC Med Res Methodol.

[15] Ward DJ (2013). Using Framework Analysis in nursing research: a worked example. J Adv Nurs.

[16] Ritchie J, Spencer L, O'Connor W, O'Connor W, Ritchie J, Spencer L (2003). Analysis: Practices, Principles and Processes. Qualitative Research Practices.

[17] Son M (2014). Barriers to Access, Disclosure, and Identification in Healthcare for Potentially Trafficked Youth in Vermont, in 142nd APHA Annual Meeting and Exposition.

[18] Taskforce on Trafficking of Women and Girls, Report of the taskforce on trafficking of women and girls. Washington DC: American Psychological Association; 2014.

[19] Baldwin S (2009). Human Trafficking Victims: At an Abortion Clinic Near You?.

[20] Baldwin SB (2011). Identification of human trafficking victims in health care settings. Health & Human Rights. Int J.

[21] HM Government, Working together to safeguard children - safeguarding children who may have been trafficked, Department for Children Schools and Families. Nottingham: Department of Children Schools and Families; 2007.

[22] Platform 51, Identifying and supporting victims of human trafficking guidance for health staff. London: Department of Health; 2013.

[23] Dottridge M (2006). Reference Guide on protecting the rights of child victims of trafficking in Europe.

[24] Family Violence Prevention Fund, Turning Pain into Power: Trafficking Survivors’ Perspectives on Early Intervention Strategies, World Childhood Foundation, San Francisco: World Childhood Foundation; 2005.

[25] International Organization for Migration, The IOM Handbook on Direct Assistance for Victims of Trafficking, International Organization for Migration. Geneva: International Organization for Migration; 2007.

[26] Isaac R, Solak J, Giardino AP. Health Care Providers’ Training Needs Related to Human Trafficking: Maximizing the Opportunity to Effectively Screen and Intervene. Journal of Applied Research on Children: Informing Policy for Children at Risk. 2011:2(1)1–32.

[27] Lederer LJ, Wetzel CA, The Health Consequences of Sex Trafficking and Their Implications for Identifying Victims in Healthcare Facilities. Annals of Health Law, 2014:23(1)61–91.

[28] London Safeguarding Children's Board (2011). London safeguarding trafficked children guidance.

[29] Patel RB, Ahn R, Burke T (2010). Human Trafficking in the Emergency Department. Western J Emerg Med.

[30] Sy E, et al. Responding to Commercially Sexually Exploited Children (CSEC): A Community Health Center’s Journey Towards Creating the First Primary Care Clinical CSEC Screening Tool in the United States. 2014.

[31] Welsh Assembly Government (2008). Safeguarding children who may have been trafficked.

[32] Borland R, Zimmerman C (2009). Caring for Trafficked Persons: Guidance for Health Providers.

[33] Dovydaitis T (2010). Human trafficking: the role of the health care provider. J Midwifery Womens Health.

[34] Ahn R (2013). Human trafficking: Review of educational resources for health professionals. Am J Prev Med.

[35] Chisolm-Straker M, Richardson LD, Cossio T (2012). Combating slavery in the 21st century: the role of emergency medicine. J Health Care Poor Underserved.

[36] Zimmerman C (2003). The Health Risks and Consequences of Trafficking in Women and Adolescents: Findings from a European Study.

[37] Macy RJ, Johns N (2011). Aftercare Services for International Sex Trafficking Survivors: Informing U.S. Service and Program Development in an Emerging Practice Area. Trauma Violence Abuse.

[38] Kung J (2014). Sex trafficking: an exploration of clinician perspectives of the type and efficacy of treatment interventions.

[39] Aron LY, Zweig JM, LS. Newmark, Comprehensive Services for Survivors of Human Trafficking: Findingsfrom Clients in Three Communities. Washington DC: Urban Institute; 2006.

[40] Cecchet SJ. The Psychological Experience of Child and Adolescent Sex Trafficking in the United States: Trauma and Resilience in Survivors in School of Psychology, Family & Community 2012, Seattle Pacific University. p. 1–130

[41] Malloch M, Warden T, Hamilton-Smith N. Care And Support for Adult Victims of Trafficking in Human Beings: A Review, Scottish Centre for Crime and Justice Research. 2012. Stirling.

[42] Hom KA, Woods SJ (2013). Trauma and its aftermath for commercially sexually exploited women as told by front-line service providers. Issues Ment Health Nurs.

[43] Baráth A, Da Victoria Lobo A, Hoxha-Beganovic R, Jaffe PD, Motus N, Szilard I, Tudorache D, Venelinova R, Weekers J. The mental health aspects of trafficking in human beings: a set of minimum standards. Budapest: International Organization for Migration; 2004

[44] Pace P. Migration and the Right to Health: A Review of European Community Law and Council of Europe Instruments, International Organisation for Migration. Geneva: International Organization for Migration; 2007.

[45] Bennett-Murphy LM (2012). Haunted: Treatment of a child survivor of human trafficking. J Infant Child Adolesc Psychother.

[46] Hardy VL, Compton KD, McPhatter VS (2013). Domestic Minor Sex Trafficking: Practice Implications for Mental Health Professionals. Aff J Women Social Work.

[47] Koleva M (2011). Psychodrama and the treatment of women victims of human trafficking: research report. Int J Psychother.

[48] Clawson HJ, et al. Study of HHS programs serving human trafficking victims 2009, U.S. Department of Health and Human Services Office of the Assistant Secretary for Planning and Evaluation: Washington DC: Department of Health and Human Services, Office of the Assistant Secretary for Planning and Evaluation.

[49] Yakushko O (2009). Human Trafficking: A review for mental health professionals. Int J Advan Counsel.

[50] Miller E (2007). Migration, sexual exploitation, and women’s health: a case report from a community health center. Violence Against Women.

[51] Abu-Ali A, Al-Bahar M (2011). Understanding child survivors of human trafficking: A micro and macro level analysis. Procedia Soc Behav Sci.

[52] European Commission, Recommendations on identification and referral to services of victims of trafficking in human beings. Strasbourg: European Commission; 2008.

[53] The Scottish Government, National guidance for child protection in Scotland. Edinburgh; The Scottish Government; 2014.

[54] Schloenhardt A, Klug B (2011). Trafficking in persons and victim health in Australia. J Law Med.

[55] Riley R (2014). Identifying and referring victims of human trafficking; training for healthcare professionals.

[56] Women’s National Commission, Still We Rise: Report from WNC Focus Groups to inform the Cross-Government Consultation “Together We Can End Violence Against Women and Girls”, Women’s National Commission. London: Women's National Commission; 2009.

[57] National Institute for Health and Care Excellence, Domestic violence and abuse: How health services, social care and the organisations they work with can respond effectively, PH50 NICE Public Health Guidance. 2014.

[58] World Health Organisation (2013). Responding to Intimate Partner Violence and Sexual Violence against Women: WHO Clinical and Policy Guidelines.

[59] Domoney J (2015). Mental health service responses to human trafficking: A qualitative study of professionals’ experiences of providing care. BMC Psychiatry.

