# Supplementary material for: Responding to the health needs of survivors of human trafficking: a systematic review
Source: BMC Health Serv Res. 2016 Jul 29;16:320. doi: 10.1186/s12913-016-1538-8 (PMC4966814; doi:10.1186/s12913-016-1538-8)
Supplement: Additional file 2: — Details of databases searched for the review. (DOCX 15 kb) [file 12913_2016_1538_MOESM2_ESM.docx]

**Databases Searched**

General Databases

1. ASSIA (Applied Social Science Index and Abstracts);
2. British Nursing Index;
3. Campbell Collaboration;
4. Cochrane Database of Systematic Reviews;
5. CINAHL (Cumulative Index of Nursing and Allied Health Literature);
6. EMBASE;
7. ERIC (Education Resources Information Centre);
8. HMIC;
9. MEDLINE;
10. Prospero;
11. PsycINFO;
12. Social Policy and Practice;
13. Social Science Citation Index;
14. Sociological Abstracts;
15. UK Clinical Research Network;

Grey Literature Databases

1. National Technical Information Service;
2. OpenGrey;
3. SCIE (Social Care Institute for Excellence);
4. Conference Proceedings Index (Science/Social Science & Humanities);
5. DART;
6. ETHOS;
7. PapersFirst;
8. ProceedingsFirst;
9. Theses Canada Portal

Websites

1. Daphne III Programme;
2. Humantrafficking.org;
3. Innocenti Project;
4. International Labour Organization;
5. International Organization for Migration;
6. La Strata International Documentation Centre;
7. NHS Evidence;
8. World Health Organization;
9. Global Alliance Against Trafficking in Women;
10. Department of Health (UK);
11. Department of Health and Human Services (USA);
12. ECPAT UK
13. Helen Bamber Foundation
14. Eaves Housing for Women
