# Supplementary material for: Responding to the health needs of survivors of human trafficking: a systematic review
Source: BMC Health Serv Res. 2016 Jul 29;16:320. doi: 10.1186/s12913-016-1538-8 (PMC4966814; doi:10.1186/s12913-016-1538-8)
Supplement: Additional file 3: — Search terms used. (DOCX 15 kb) [file 12913_2016_1538_MOESM3_ESM.docx]

**Search Terms Used**

1. “human trafficking” OR “people trafficking” OR “sex trafficking” OR “child trafficking” OR “trafficking in people” OR “victim of trafficking” OR “survivor of trafficking” OR “trafficking for sexual exploitation” OR “trafficking for labour exploitation” OR “trafficked people” OR “trafficked women” OR “trafficked men” OR “trafficked children” OR “trafficked person$” OR “domestic servitude” OR “domestic slavery” OR “forced labour” OR “forced labor” OR “forced prostitution” OR “sexual slavery” OR “sex slave”
2. Health$ OR service OR care OR medic$ OR therap$ OR hospital OR clinic OR reprod$ OR abort$ OR contracept$ OR pregnan$ OR terminat$ OR family planning OR emergency OR psycho$ OR psychiatr$ OR antenatal OR neonatal OR obstetr$ OR gynecol$ OR gynaecol$ OR drug OR alcohol OR substance OR trauma$ OR injury OR accident OR welfare OR safeguard$ OR Health Services/ OR clinician OR community health worker OR dentist OR doctor OR general practitioner OR health provider OR health visitor OR nurs$ OR pharmacist OR physician OR healthcare worker OR health professional OR healthcare professional OR Health personnel/
3. identif$ OR recogni$ OR respon$ OR disclos$ OR screen$ OR assess$ OR aftercare OR treat$ OR examin$ OR monitor$ OR protect$ OR Outreach OR assist$ OR Interven$ OR prevent$ OR notifi$ OR help OR Harm reduction OR support OR advi$e OR advoca$ OR counseling OR counselling OR hotline OR helpline OR Refer$ Or coordinat$ OR information sharing OR training OR awareness OR knowledge OR guidance OR guide$ OR protocol OR tool$ OR practice OR manual OR curric$ OR educ$ OR case study OR policy OR strateg$ OR approach OR evaluat$ OR feasibility OR pilot OR validat$ OR efficacy OR efficient OR effective OR impact OR Disclosure/ OR Mass Screening/ OR Harm reduction/ OR Referral and Consultation/ OR Attitude of health professional/ OR Clinical competence/ OR Education, Medical, Continuing/ OR General Practice/education/ OR Physician’s Practice Patterns/ OR Quality Assurance, Health Care/ OR Evaluation Studies/ OR Feasibility Studies/ OR Outcome Assessment (Healthcare)/ OR Outcome and Process Assessment (Health Care)/ OR Pilot Projects/ OR Project Evaluation/ OR Treatment Outcome/ OR Validation Studies/ OR Professional-Patient Relations/ OR Qualitative research/ OR Heath services accessibility/ OR Patient acceptance of healthcare/
4. Protein OR membrane OR call
5. (1 AND (2 OR 3)) NOT 4
