# Supplementary material for: Responding to the health needs of survivors of human trafficking: a systematic review
Source: BMC Health Serv Res. 2016 Jul 29;16:320. doi: 10.1186/s12913-016-1538-8 (PMC4966814; doi:10.1186/s12913-016-1538-8)
Supplement: Additional file 4: — Quality appraisal checklist. (DOCX 15 kb) [file 12913_2016_1538_MOESM4_ESM.docx]

**Quality Appraisal Checklists (adapted from the Joanna Briggs Institute Quality Appraisal Checklists).**

Narrative, opinion, policy, or guidance documents

1. Is the source of opinion clearly identified?
2. Does the source of opinion have standing in its field of expertise?
3. Are the interests of patients/clients the central focus of the opinion?
4. Is the opinion’s basis in logic/experience clearly argued?
5. Is the argument developed analytically?
6. Is there reference to literature/evidence?
7. Is any incongruence with literature/evidence clearly stated?

Reviews

1. Is the review question clearly and explicitly stated?
2. Is the search strategy appropriate?
3. Are the sources of studies adequate?
4. Are the inclusion criteria appropriate for the review question?
5. Are the criteria for appraising the studies appropriate?
6. Is critical appraisal conducted by two or more reviewers independently?
7. Are there methods used to minimise error in data extraction?
8. Are the methods used to combine studies appropriate?
9. Are the recommendations supported by the reported data?
10. Are the specific directives for new research appropriate?

Qualitative research

1. Is there congruity between the philosophical perspective and research methodology?
2. Is there congruity between the research methodology and the research questions/objectives?
3. Is there congruity between the research methodology and the methods used to collect data?
4. Is there congruity between research methodology and the representation and analysis of data?
5. Is there congruity between research methodology and the interpretation of results?
6. Is a statement presented locating research culturally or theoretically?
7. Is the influence of the researcher on the research – and vice versa- addressed?
8. Do the people collecting data have training on conducting research with trafficked people?
9. Is research conducted in collaboration with support organisations/referral and support information provided?
10. Is evidence of ethical approval presented?
11. Are participants, and their voices, adequately represented?
12. Do conclusions flow from the analysis/interpretation of the data?
13. Is the repeatability or reliability of results discussed?
14. Is the validity or trustworthiness of the results discussed?

Cohort and case control studies

1. Do the people collecting data have training on conducting research with trafficked people?
2. Is research conducted in collaboration with support organisations/referral and support information provided?
3. Is the sample representative of patients in the population as a whole?
4. Are patients at a similar point in the course of their illness/condition?
5. Is bias minimised in relation to the selection of cases and controls?
6. Are confounding factors identified and strategies to deal with them described?
7. Are outcomes assessed using objective criteria?
8. Is follow up carried out over a sufficient time period?
9. Are the outcomes of people who withdrew from the study described and included in the analysis?
10. Are outcomes measured in a reliable way?
11. Is appropriate statistical analysis used?

Descriptive and case series studies

1. Do the people collecting data have training on conducting research with trafficked people?
2. Is research conducted in collaboration with support organisations/referral and support information provided?
3. Is the study based on a random or pseudo-random sample?
4. Are the criteria for inclusion in the sample clearly defined?
5. Are confounding factors identified and strategies to deal with them described?
6. Are outcomes assessed using objective criteria?
7. If comparisons are being made, are there sufficient descriptions of the groups?
8. Is follow up carried out over a sufficient time period?
9. Are the outcomes of people who withdrew from the study described and included in the analysis?
10. Are outcomes measured in a reliable way?
11. Is appropriate statistical analysis used?
