# Supplementary material for: Responding to the health needs of survivors of human trafficking: a systematic review
Source: BMC Health Serv Res. 2016 Jul 29;16:320. doi: 10.1186/s12913-016-1538-8 (PMC4966814; doi:10.1186/s12913-016-1538-8)
Supplement: Additional file 5: Table S1. — Characteristics and quality scores of included studies. (DOCX 28 kb) [file 12913_2016_1538_MOESM5_ESM.docx]

***Table 1: Characteristics and quality scores of included studies.***

|  |  | **Study Characteristics** |  |  |  |  | **Appraisal** |
| --- | --- | --- | --- | --- | --- | --- | --- |
|  |  |  |  |  |  |  |  |
| **Source** | **Study Type** | **Study Aims** | **Study country** | **Type of Health Setting** | **Method** | **Type of Trafficking** | **Appraisal** |
| Abu-Ali & Ali-Bahar (2011) | Narrative | To discuss the social aspects of child trafficking and the psychological consequences experienced. | United Arab Emirates | Mental Health - Psychotherapy | Narrative | Not Specified | 11/14 |
| Ahn *et al*. (2013) | Review | To review identified educational resources about human trafficking for healthcare providers. | United States of America | General health | Review | Not Specified | 18/20 |
| Aron *et al*. (2006) | Primary Research | To learn more about the victim services being provided by the Office for Victims of Crime for victims of human trafficking. | United States of America | General health | In-depth interviews with 32 female survivors and 2 male survivors. | Labour trafficking including domestic servitude; Sex trafficking including forced sex work and servile marriage) | 17/28 |
| Baldwin *et al.* (2009) | Primary Research | To characterise human trafficking victims interactions with the healthcare systems in order to facilitate identification. | United States of America | General health | Semi-Structured interview with 6 survivors and 12 key informants | Not Specified | 22/28 |
| Baldwin *et al*. (2011) | Primary Research | To explore and characterise encounters in healthcare settings by victims of human trafficking. | United States of America | General health | Semi-Structured Interviews with 6 key informants and 12 female survivors. | Domestic Servitude; Sex Trafficking | 13/14 |
| Barath *et al.* (2004) | Guidance | Set of minimum standards for mental health provision for victims of human trafficking. | Switzerland | Mental Health | Guidance | Not Specified | 12/14 |
| Bennett-Murphy (2012) | Narrative | To examine challenges in the psychological treatment of child survivors of human trafficking. | United States of America | Mental Health- Psychotherapy | Narrative | Child Trafficking | 14/14 |
| Borland & Zimmerman (2009) | Guidance | To provide practical, non-clinical guidance to help healthcare providers recognise and consider approaches to providing care for victims of human trafficking. | Switzerland | General health | Guidance | Not Specified | 14/14 |
| Cecchett (2012) | Primary Research | To assess the ability of child sex trafficking survivors to survive, leave the sex trade and reintegrate into the community. | United States of America | Mental Health | In-depth Interviews with 6 female Survivors | Child Sex trafficking – Sex Work | 17/22 |
| Chisolm-Straker *et al*. (2012) | Primary Research | To develop and pilot a training intervention for emergency providers on human trafficking and how to identify and treat survivors. | United States of America | Emergency Department | Cross Sectional Survey – Training Evaluation | Not Specified | 13/14 |
| Chisolm-Straker et al. (2014) | Primary Research | To describe the healthcare experiences of those being trafficked. | United States of America | Emergency Department | Cross Sectional Survey with 173 male and female Survivors | Sex and Forced Labour Trafficking | 11/22 |
| Clawson *et al*. (2009) | Primary Research | To provide a more in-depth understanding of human trafficking, victims and services provided to meet their needs. | United States of America | General health | Qualitative Interviews with 341 service providers across 11 USA states. | Not Specified | 19/28 |
| European Commission  (2008) | Guidance | Recommendations on identification and referral to services for victims of human trafficking. | European Union | General health | Guidance | Not Specified | 11/14 |
| Dottridge (2006) | Guidance | Guidance aimed to assist implementation of the UNICEF Guidelines on Protection of the Rights of Child Victims of Trafficking. | European Union | General health | Guidance | Child Trafficking | 13/14 |
| Dovydaitis (2010) | Narrative | To provide clinicians with knowledge on human trafficking, and to give specific tools that they may use to assist victims in clinical settings. | United States of America | General health | Case Study (Domestic Servitude) | Not specified | 11/14 |
| European Union Agency For Fundamental Rights (2009) | Guidance | To outline the challenges, perspectives and good practices on child trafficking in the European Union. | European Union | General health | Guidance | Child Trafficking | 13/14 |
| European Parliament (2011) | Policy | Directive on preventing and combating trafficking in human beings and protecting its victims. | European Union | General health | Policy | Not Specified | 12/14 |
| Family Violence Prevention Fund (2005) | Primary Research | To determine if healthcare settings were appropriate places to screen and intervene with trafficked women and children and to recommend strategies to improve the healthcare of trafficked victims. | United States of America | General health | In-depth interviews with 19 female survivors and 2 male survivors. | Labour trafficking including domestic servitude; Sex trafficking including forced sex work and servile marriage) | 25/28 |
| Hardy *et al*. (2013) | Narrative | Discussion of the possible effects of domestic minor sex trafficking, implications for intervention and future research. | United States of America | Mental Health | Narrative | Domestic minor sex trafficking | 13/14 |
| HM Government (DCSF) (2008) | Guidance | Practice guidance outlining reasons for child trafficking, the methods used by traffickers, the roles and functions of relevant agencies and how practitioners should follow procedures to ensure the safety and wellbeing of children. | United Kingdom | General health | Guidance | Child Trafficking. | 14/14 |
| Hom & Woods (2013) | Primary Research | To describe the experiences of trauma and its aftermath for women who have experienced commercial sexual exploitation as told by front line workers. | United States of America | General health | Semi-Structured Interviews with 6 front line service providers. | Sex trafficking; forced sex work. | 23/28 |
| International Organisation for Migration (2007) | Review | Review of European community law and council of Europe instruments in relation to migrants’ right to health. | Switzerland | General health | Review | Not Specified | 14/20 |
| Isaac *et al.* (2007) | Narrative | To explore health care issues associated with victims of human trafficking and the training that front line physicians and nurses require to screen and identify. | United States of America | General health | Narrative | Not Specified | 13/14 |
| Koleva (2011) | Narrative | To explore how psychodrama can respond to the therapeutic needs of female victims of human trafficking. | Netherlands | Mental Health - Psychotherapy | Case Study | Not Specified | 11/14 |
| Kung (2014) | Primary Research | To investigate clinicians perspectives on the type of clinical interventions used in therapy with sex trafficking victims. | United States of America | Mental Health | In-depth interviews with 11 clinicians. | Sex Trafficking | 21/28 |
| Lederer & Wetzel (2014) | Primary Research | To explore the health consequences and experience of women and girls trafficked in the United States for sex. | United States of America | General health | Cross Sectional Survey, 107 semi-structured interviews with survivors, focus groups. | Domestic Sex Trafficking. | 30/48 |
| London Safeguarding Children Board (2011) | Guidance | To provide guidance to professionals and volunteers from all agencies in safeguarding and promoting the welfare of trafficked and exploited children. | United Kingdom | General health | Guidance | Not Specified | 14/14 |
| Macy & Johns (2011) | Review | To review the needs of and services for international survivors of sex trafficking into the United States. | United States of America | After Care Services | Systematic Review | Sex Trafficking | 14/20 |
| Miller *et al*. (2007) | Primary Research | To describe the vulnerabilities to forced prostitution as a result of trafficking and the challenges to providing comprehensive responses to health needs. | United States of America | Community Health | Case Study – Forced Sex Work | Sex Trafficking – Forced Sex Work | 14/14 |
| Malloch *et al.* (2012) | Review | A review of care and support for adult victims of human trafficking. | United Kingdom | General health | Review | Not Specified | 10/20 |
| Pace (2007) | Guidance | Guidance on the provision of assistance to victims of human trafficking. | Switzerland | General health | Guidance | Not Specified | 14/14 |
| Patel *et al*. (2010) | Primary Research | To highlight a case example of a human trafficking patient in the complexities of identification in healthcare. | United States of America | Emergency Department | Case Study | Sex trafficking. | 14/14 |
| Platform 51 (2013) | Guidance | Guidance for healthcare staff on identifying and supporting victims of human trafficking. | United Kingdom | General health | Guidance | Not Specified | 12/14 |
| Riley (2013) | Primary Research | To pilot a non-paper training programme for healthcare professionals on human trafficking in the United Kingdom. | United Kingdom | Primary Healthcare | Cross Sectional Survey – Training Evaluation | Not Specified | 13/22 |
| Son *et al.* (2014) | Primary Research | To identify barriers to access, disclosure and identification in healthcare settings for potential trafficked youth in Vermont. | United States of America | Drop-In Centres | Cross Sectional Survey with Healthcare Providers and At Risk Youth | Not Specified | 5/22 |
| The Scottish Government (2014) | Guidance | National guidance on child protection in Scotland 2014. | United Kingdom | General health | Guidance | Child Trafficking | 13/14 |
| Scholenhardt & Klug (2011) | Narrative | To explore the health problems experienced by victims of human trafficking in Australia and analyse the domestic support schemes established to assist victims. | Australia | General health | Narrative | Not Specified | 11/14 |
| Sy *et al.* (2014) | Primary Research | To discuss the need for and development process of a screening tool for commercially sexually exploited children. | United States of America | Community Health | Interviews and Focus Groups with Survivors and Healthcare Staff | Sexual Exploitation; Child Trafficking. | 17/28 |
| Trafficking Taskforce APA (2014) | Review | To raise awareness amongst psychologists of human trafficking and to make recommendations to enhance research, education/training and policy from psychologists in relation to human trafficking. | United States of America | Mental Health | Review | Not Specified | 16/20 |
| Taskforce on the Health Aspects of Violence Against Women & Children (2010) | Guidance | Health taskforce recommendations in the role of the UK National Health Service in harmful traditional practices and human trafficking subgroup. | United Kingdom | General health | Taskforce | Not Specified | 13/14 |
| Welsh Assembly Government (2008) | Guidance | To provide good practice guidance to professionals and volunteers from all agencies to help them safeguard children who have been trafficked. | United Kingdom | General health | Guidance | Child Trafficking. | 12/14 |
| Women’s National Commission (2009) | Primary Research | Recommendations on the prevention, provision and protection from violence against women and girls. | United Kingdom | General health | Focus groups with female survivors of violence including trafficking. | Not Specified | 22/20 |
| Yakusho (2009) | Review | To review current research on human trafficking for mental health practitioners and scholars and suggestions for treatment. | United States of America | Mental Health | Review | Not Specified | 12/20 |
| Zimmerman *et al*. (2003) | Primary Research | To highlight the health risks and consequences of trafficking in women and to provide information on health needs. | United Kingdom | General health | In-depth interviews with 28 female survivors and 107 key informants. | Forced sex work; Domestic Labour | 26/28 |
