# Supplementary material for: Responding to the health needs of survivors of human trafficking: a systematic review
Source: BMC Health Serv Res. 2016 Jul 29;16:320. doi: 10.1186/s12913-016-1538-8 (PMC4966814; doi:10.1186/s12913-016-1538-8)
Supplement: Additional file 6: Table S2. — Potential Identifiers for Adult and Child Victims of Human Trafficking. (DOCX 30 kb) [file 12913_2016_1538_MOESM6_ESM.docx]

**Table 2: Potential Identifiers for Adult and Child Victims of Human Trafficking**

|  |  |  |  |  |  |  | ***Papers Reporting Identifiers for Human Trafficking*** | | | | | | | | | | | | | | |  | |  | |  | |  |
| --- | --- | --- | --- | --- | --- | --- | --- | --- | --- | --- | --- | --- | --- | --- | --- | --- | --- | --- | --- | --- | --- | --- | --- | --- | --- | --- | --- | --- |
|  | APA (2014) | Baldwin et al. (2009) | Baldwin et al. (2011) | DSCF (2008) | Platform 51 (2013) | Dottridge (UNICEF) (2006) | Dovydaitis (2010) | | Family Violence Prevention Fund (2005) | | Pace  (2007) | | Isaac et al. (2007) | Lederer & Wetzel (2014) | | LSCB (2011) | | Patel *et al*. (2010) | | Son *et al.* (2014) | | Sy *et al.* (2014) | | Welsh Assembly (2008) | | Borland &Zimmerman (2009) | |  |
| Indicators of Physical Abuse | ***🗸*** | ***🗸*** | ***🗸*** | ***🗸*** | ***🗸*** | ***🗸*** | ***🗸*** | | ***🗸*** | | ***🗸*** | | ***🗸*** | ***🗸*** | | ***🗸*** | | ***🗸*** | | ***🗸*** | |  | | ***🗸*** | | ***🗸*** | |  |
| Indicators of Psychological Abuse | ***🗸*** | ***🗸*** | ***🗸*** |  | ***🗸*** | ***🗸*** | ***🗸*** | |  | | ***🗸*** | | ***🗸*** | ***🗸*** | | ***🗸*** | |  | | ***🗸*** | |  | | ***🗸*** | | ***🗸*** | |  |
| Indicators of Sexual Abuse | ***🗸*** | ***🗸*** | ***🗸*** | ***🗸*** |  | ***🗸*** | ***🗸*** | | ***🗸*** | | ***🗸*** | | ***🗸*** | ***🗸*** | | ***🗸*** | |  | | ***🗸*** | | ***🗸*** | | ***🗸*** | | ***🗸*** | |  |
| Indicators of Neglect |  |  |  | ***🗸*** | ***🗸*** |  | ***🗸*** | | ***🗸*** | |  | |  |  | | ***🗸*** | |  | |  | |  | | ***🗸*** | | ***🗸*** | |  |
| Substance Misuse | ***🗸*** |  |  | ***🗸*** |  |  |  | |  | |  | |  | ***🗸*** | |  | |  | |  | |  | | ***🗸*** | | ***🗸*** | |  |
| Lack of Official Documents/False Documents | ***🗸*** |  |  | ***🗸*** | ***🗸*** | ***🗸*** | ***🗸*** | | ***🗸*** | | ***🗸*** | | ***🗸*** |  | | ***🗸*** | |  | |  | |  | | ***🗸*** | |  | |  |
| Accompanied by Controlling Companion | ***🗸*** | ***🗸*** |  | ***🗸*** | ***🗸*** |  | ***🗸*** | | ***🗸*** | | ***🗸*** | | ***🗸*** | ***🗸*** | | ***🗸*** | | ***🗸*** | | ***🗸*** | |  | | ***🗸*** | |  | |  |
| Poor Engagement/Goes missing |  |  |  | ***🗸*** |  |  |  | |  | |  | |  |  | |  | |  | |  | |  | | ***🗸*** | |  | |  |
| Poor Living and Working Conditions | ***🗸*** |  |  | ***🗸*** |  | ***🗸*** |  | |  | |  | |  |  | | ***🗸*** | |  | |  | |  | | ***🗸*** | | ***🗸*** | |  |
| Lack of Social Network |  |  |  |  |  |  |  | |  | |  | |  |  | |  | |  | | ***🗸*** | |  | |  | |  | |  |
| Unable to speak local language |  | ***🗸*** | ***🗸*** |  | ***🗸*** |  | ***🗸*** | | ***🗸*** | | ***🗸*** | | ***🗸*** | ***🗸*** | |  | | ***🗸*** | |  | |  | |  | |  | |  |
| Excessive fear of deportation/legal status |  |  |  | ***🗸*** |  |  | ***🗸*** | |  | |  | |  |  | | ***🗸*** | |  | |  | |  | | ***🗸*** | | ***🗸*** | |  |
| Fear of Authority Figures |  |  |  | ***🗸*** |  |  | ***🗸*** | | ***🗸*** | | ***🗸*** | | ***🗸*** |  | | ***🗸*** | |  | |  | |  | | ***🗸*** | | ***🗸*** | |  |
| Presence of Tattoo's Marking Property | ***🗸*** |  |  |  |  |  |  | |  | | ***🗸*** | | ***🗸*** | ***🗸*** | |  | |  | | ***🗸*** | |  | |  | |  | |  |
| Moves location frequently |  |  |  |  | ***🗸*** |  |  | |  | |  | |  |  | |  | |  | |  | |  | | ***🗸*** | | ***🗸*** | |  |
| Limited personal freedom |  |  |  |  |  |  | ***🗸*** | |  | |  | |  |  | | ***🗸*** | |  | |  | |  | | ***🗸*** | | ***🗸*** | |  |
| Entered Country Illegally |  |  |  | ***🗸*** |  |  |  | |  | |  | | ***🗸*** |  | | ***🗸*** | | ***🗸*** | |  | |  | | ***🗸*** | |  | |  |
| Not registered with GP or School |  |  |  | ***🗸*** | ***🗸*** |  |  | |  | |  | |  |  | | ***🗸*** | |  | |  | | ***🗸*** | | ***🗸*** | |  | |  |
| Inconsistencies in Presentation |  |  |  | ***🗸*** | ***🗸*** |  | ***🗸*** | | ***🗸*** | | ***🗸*** | |  |  | | ***🗸*** | | ***🗸*** | | ***🗸*** | |  | | ***🗸*** | |  | |  |
| Medical Treatment paid for in cash |  |  | ***🗸*** |  |  |  |  | |  | |  | |  |  | |  | |  | | ***🗸*** | |  | |  | |  | |  |
|  |  |  |  |  |  |  |  | |  | |  | |  |  | |  | |  | |  | |  | |  | |  | |  |
|  |  |  |  |  |  |  | **Additional Child Identifiers Indicated** | | | | | | |  | |  | |  | |  | |  | |  | |  | |  |
| Possesses money or goods not accounted for |  |  |  | ***🗸*** |  |  |  |  | |  | |  | | |  | | ***🗸*** | |  | | ***🗸*** | |  | | ***🗸*** | |  | |
| Has a mobile phone but no other possessions or money |  |  |  | ***🗸*** |  |  |  |  | |  | |  | | |  | | ***🗸*** | |  | | ***🗸*** | |  | | ***🗸*** | |  | |
| Receives unexplained phone-calls whilst in setting |  |  |  | ***🗸*** |  |  |  |  | |  | |  | | |  | | ***🗸*** | |  | | ***🗸*** | |  | | ***🗸*** | |  | |
| Is one of a number of unrelated children living at an address |  |  |  | ***🗸*** |  |  |  |  | |  | |  | | |  | | ***🗸*** | |  | | ***🞍*** | |  | | ***🞍*** | |  | |
| Shows signs of maturity or confidence beyond child's age |  |  |  | ***🗸*** |  |  |  |  | |  | |  | | |  | | ***🗸*** | |  | |  | |  | | ***🗸*** | |  | |
| Truancy from school/ Goes missing from Local Authority Care |  |  |  | ***🗸*** | ***🗸*** |  |  |  | |  | |  | | |  | | ***🗸*** | |  | | ***🗸*** | | ***🗸*** | | ***🗸*** | |  | |
| Is known to beg for money /involved in Criminal Activity |  |  |  | ***🗸*** |  | ***🗸*** |  |  | |  | |  | | |  | | ***🗸*** | |  | |  | |  | |  | |  | |
| Not Living with Parents/Relatives/poor relationship with adult carer |  |  |  | ***🗸*** |  | ***🗸*** |  |  | |  | |  | | |  | | ***🗸*** | |  | |  | |  | | ***🗸*** | |  | |
| Under pressure to pay debts |  |  |  | ***🗸*** |  |  |  |  | |  | |  | | |  | | ***🗸*** | |  | |  | |  | | ***🗸*** | |  | |
| Travel arrangements paid and organised by someone else |  |  |  | ***🗸*** |  | ***🗸*** |  |  | |  | |  | | |  | | ***🗸*** | |  | |  | |  | | ***🗸*** | |  | |
| Has a prepared story similar to other children |  |  |  | ***🗸*** |  | ***🗸*** |  |  | |  | |  | | |  | | ***🗸*** | |  | |  | |  | | ***🗸*** | |  | |
| Dresses Inappropriately for the Weather/Setting |  |  |  | ***🗸*** |  |  |  |  | |  | |  | | |  | | ***🗸*** | |  | | ***🗸*** | |  | |  | |  | |
